# Clinical control study of traditional Chinese medicine hot compress combined with traction in the treatment of cervical spondylotic radiculopathy: Study protocol

**DOI:** 10.1097/MD.0000000000023880

**Published:** 2021-01-29

**Authors:** Xing Ding, Jinze Wu, Qixing Shen, Jinhai Xu, Wen Mo

**Affiliations:** Department of Orthopaedics, Longhua Hospital, Shanghai University of Traditional Chinese Medicine, No. 725, Wanping South Road, Fenglin Street, Xuhui District, Shanghai, China.

**Keywords:** cervical spondylotic radiculopathy (CSR), hot compress, traction

## Abstract

Cervical spondylotic radiculopathy (CSR) is the most common type of cervical spondylosis, accounting for about 60% of the incidence of cervical spondylosis. Both cervical traction and traditional Chinese medicine hot compress are common and effective treatment for CSR. This study will be performed to investigate the effect of a combination of cervical traction and traditional Chinese medicine hot compress on CSR. In this non-blinded, randomized controlled trial, 100 eligible patients will be randomly divided into a treatment group (intermittent cervical traction combines with traditional Chinese medicine hot compress) and a control group (intermittent cervical traction combined with hot compresses). Before and after the intervention, the Visual Analog Scale score, Neck Disability Index score, and 20-score scale of symptoms will be evaluated at baseline and at 7, 14, 21, and 28 days. During the treatment period, any signs of acute adverse events, such as paralysis of aggravated pain, nausea, dizzy, and even syncope, will be recorded at each visit. Although intermittent cervical traction and traditional Chinese medicine hot compress have been used in the treatment of CSR in China for many years, there is no consensus on its effectiveness of combination therapy. This experiment will provide convincing evidence of the efficacy of intermittent cervical traction combined with traditional Chinese medicine hot compress in the treatment of CSR.

## 1 Introduction

Cervical spondylosis is a syndrome in which degenerative changes or secondary changes of cervical intervertebral discs stimulate nerve roots, spinal cord, vertebral arteries, or sympathetic nerves, etc., which cause related symptoms and signs.^[1]^ Cervical spondylotic radiculopathy (CSR) is the most common type of cervical spondylosis, accounting for about 60% of the incidence of cervical spondylosis.^[2]^ The treatment methods of CSR are mainly divided into surgical treatment and conservative treatment. Surgical treatment is not considered as the first choice for CSR. At present, there are a lot of nondrug conservative treatment methods, such as acupuncture, massage, Daoyin exercises, traditional Chinese medicine compresses, low-frequency therapy, microwave, etc., and they all have certain treatments for CSR. In the literatures,^[3]^ the clinical efficacy of conservative treatment and surgical treatment is equivalent. Therefore, it is great significant to explore how to use the optimal combination of conservative treatments to improve the neck pain and radicular symptoms of patients with radiculopathy, and alleviate the progress of cervical spondylosis.

Cervical traction is a common and effective treatment for CSR. Traction relieves the stimulation and compression of nerve roots according to improving the mechanical balance of the cervical spine, changing the relationship between disc protrusions or osteophytes and surrounding tissues. What the functions of intermittent traction are concluded^[4–6]^:

1. The force of intermittent traction is greater than the continuous traction, which is more conducive to stretch the intervertebral space and intervertebral foramen and relieve symptoms.
2. Intermittent traction can also cause the neck muscles to have a rhythmic alternating movement of tension and relaxation, resulting in a local massage effect, which is more conducive to relieve neck muscle spasm and eliminate inflammation around the nerve root.
3. Intermittent traction can restore muscles and blood vessels, and fewer symptoms such as headaches and nausea after traction, reduce the long-term direct compression of local muscles and blood vessels by the traction belt during traction, and reduce reflex caused by muscle and vasospasm. It is more likely to be accepted by patients.
4. In the process of intermittent traction, the muscles are relaxed and combined with work and rest, which reduces the possibility of continuous traction to the neck and back muscles and better relieves muscle spasms.

Traditional Chinese medicine hot compress is to directly place the herbal hot pack directly on the neck, exerting the thermal effect, heating up local tissues, expanding capillaries, accelerating blood circulation, improving local metabolism, and achieving the effects of anti-inflammatory, swelling, and reflex muscle spasms. It relaxes the muscles, relieves fatigue and pain,^[7]^ and restores the balance of the dynamic system. And hot compress is with the help of the warming effect to bring the effective ingredients of traditional Chinese medicine into the meridians and collaterals directly to the location of disease, and form a higher concentration in the neck area, fully synergizing the warming and pharmacological effects, so as to achieve the effects of warming the meridian, promoting blood circulation and relieving pain. This project intends to conduct a prospective randomized controlled trial of herbal hot compress combined with traction for the treatment of CSR patients to verify the effectiveness and safety of hot compress combined with traction for CSR treatment.

## 2 Method/design

**Figure 1:**
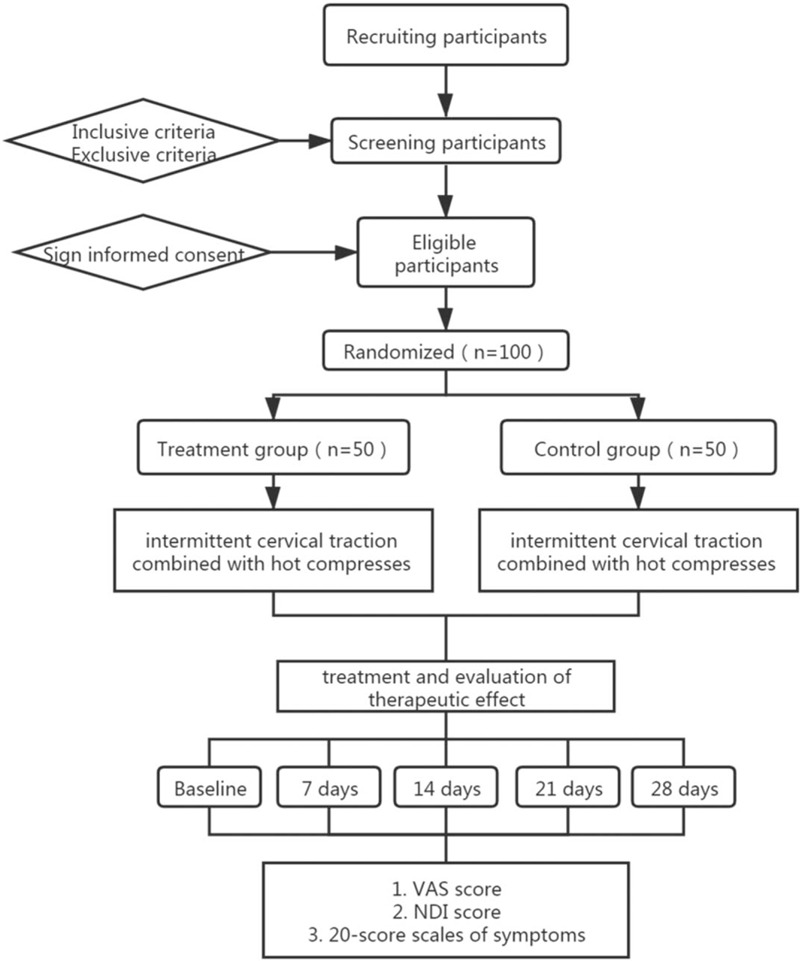
Consort flow diagram.

This study is a prospective randomized controlled clinical trial. The purpose of the proposed study is to investigate the efficacy of traditional Chinese medicine compresses combined with intermittent traction on CSR. The Principal Investigator (PI) is responsible for the entire project and organizes the steering committee meeting. The independent steering committee will be responsible for the safety of participants, meetings, recruitment and follow-up of participants, and quality control. The coordination center is responsible for communicating agreement changes and providing materials. The trial includes a 4-week treatment period and a 4-week follow-up period. After randomization, the patients will receive 2 courses of treatment within 4 weeks. Efficacy evaluations will be performed 7 days after the start of treatment, 14 days after treatment, 21 days after treatment, and 28 days after treatment (Fig. 1; Additional File 1).

### 2.1 Eligibility criteria

#### 2.1.1 Inclusion criteria

1. Meet the criteria for CSR;
2. Informed consent and signed informed consent form;
3. Be between 18 to 70 years old;
4. Patients who voluntarily accept the treatment plan of this study.

#### 2.1.2 Exclusion criteria

1. Patients who do not meet the diagnostic criteria, and patients who are under 18 years old or over 70 years old;
2. Those who are allergic to traditional Chinese medicine;
3. Women during pregnancy and lactation;
4. Patients who have undergone cervical spine surgery or have a history of neck trauma;
5. Patients with serious primary diseases or mental illnesses such as heart, brain, liver, kidney and hematopoietic system;
6. Patients with skin diseases and infectious diseases;
7. A history of diabetes and poor blood sugar control;
8. Patients whose condition is serious and are not suitable for conservative treatment or who require surgical treatment;
9. Patients who have received other treatments, which may affect the observation of treatment effect indicators in this study.

Participants in the patient recruitment program will be recruited from Longhua Hospital affiliated to Shanghai University of Traditional Chinese Medicine. The expected participants will be interviewed by the coordinator and notified of their eligibility criteria and procedures. Those who are eligible and willing to participate in the study will first be screened through a baseline assessment, and then diagnosed based on clinical manifestations, physical examination, and imaging. Participants will be told that participation in the trial is absolutely voluntary and can withdraw from the trial at any time. If you exit, the collected data will not be deleted and will be used for final analysis. A data compilation table containing all variables of interest and all potential risks will be completed by the corresponding research center. The information obtained will be stored in an electronic database for subsequent statistical analysis. Recruitment will start in September 2020 and is expected to end in December 2020. The final follow-up work for all participants will be completed on January 31, 2021. See Figure 1 for the participant processing and evaluation timeline.

#### 2.1.3 Ethics approval and consent to participate

This study will be conducted in accordance with the principles of the Helsinki Clinical Research Declaration.^[8]^ The trial protocol has been approved by the Research Ethics Committee of Longhua Hospital affiliated to Shanghai University of Traditional Chinese Medicine (2020LCSY059). All participants will have enough time to make a decision to sign the consent form before the study.^[9,10]^ This agreement has been registered on Clinical Trials (ClinicalTrials.gov, ID: ChiCTR2000037976).

#### 2.1.4 Intervention

The control group will be treated with intermittent cervical traction combined with hot compresses (device name: intelligent warm traction system, model YK-60000D), and the traction angle will be 10° to 15°; its starting mass will be 3 to 6 kg, according to the condition and the patient's The degree of tolerance can gradually increase, up to 8 to 12 kg; the traction time is 90 seconds, and the intermittent time is 10 seconds; the traction is used for hot compress treatment with an instrument matching hot compress bag, the temperature is controlled at 40 to 50°C, and the neck is applied externally. Once every day, 20 minutes times, a week of treatment for 5 days, 14 days as a course of treatment, a total of 2 courses of treatment; curative effect evaluation on 7 days, 14 days, 21 days, and 28 days after the start of treatment.

**Figure 2:**
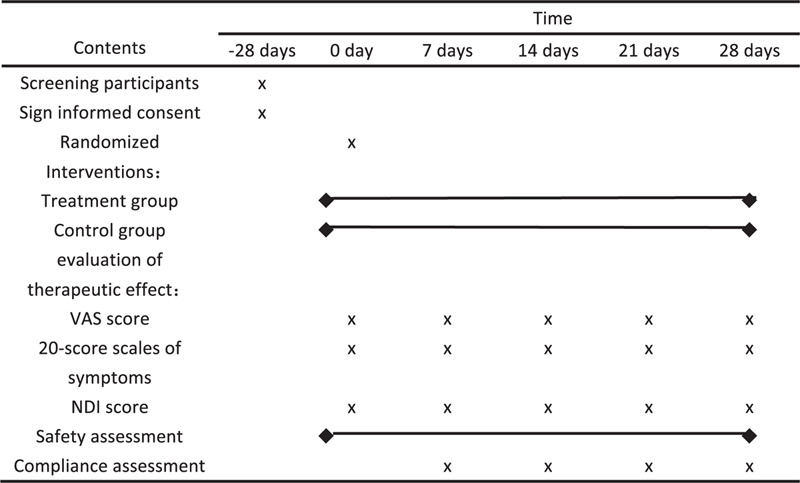
Baseline and follow-up assessments.

The intervention group will be treated with intermittent cervical traction combines with traditional Chinese medicine hot compress, and the traction method is the same as the control group. Hot compress herbs include: Corydalis, Tougucao, Frankincense, Myrrh, Angelica, Sichuan pepper, Safflower, Ligusticum chuanxiong, Licorice, Weilingxian, Parsnip, Angelica dahurica, first soak in appropriate amount of warm water and steam it in a steamer for 10 minutes. The temperature of hot pack is controlled at 40 to 50°C, and applies traditional Chinese medicine to the neck. Once a day, 20 minutes times, a week of treatment for 5 days, 14 days as the efficacy evaluation, 7 days, 14 days, 21 days, and 28 days after the start of treatment (Fig. 2; Additional File 2).

#### 2.1.5 Randomization and allocation

After screening, patients will be randomly divided into 2 groups with a 1:1 allocation ratio. Randomization will be based on the Statistical Social Science Software Package (SPSS) software (version 21.0), generating random numbers, and patients will be randomly divided into treatment group (50 cases) and control group (50 cases).

#### 2.1.6 Outcome measurement

Pain is one of the most common reasons for patients with CSR to see a doctor. Therefore, the Visual Analog Pain Scale (VAS) will be chosen as the main outcome measure for the assessment of the neck and upper limbs. The VAS score is a reliable and effective method of pain measurement. There is a 100 mm long horizontal line. The left side records “no pain” (score 0), and the right side records “pain is severe and intolerable” (score 10). The patient is required to place a mark corresponding to its current pain level on the horizontal line (the most severe pain at rest and during exercise),^[11]^ and then determine the VAS score by measurement.

Neck Disability Index (NDI)^[12]^ is one of the most commonly used indexes to evaluate patients neck dysfunction. The NDI score includes 4 subjective symptoms (neck pain intensity, headache, concentration, sleep) and 6 items related to activities of daily living (weightlifting, work, driving or cycling, entertainment, personal care, reading). Each item has 6 levels of responses, each with a score of 0 to 5, 0 means no disability, 5 means severe disability. The lowest total NDI score is 0 points, and the highest is 50 points; the higher the score, the more serious the degree of dysfunction. This study adopted all dimensions of NDI score.

Tanaka Yasuhisa Cervical Spondylopathy Symptom Scale is the “Nerve Root Cervical Spondylopathy Symptom Scale” proposed by the Japanese scholar Hisasu Tanaka. It compares the improvement index before and after treatment for evaluation: improvement rate = (points after treatment-points before treatment)/(20 -Points before treatment)∗100%.

#### 2.1.7 Assess safety

To ensure the safety of participants, the data collection process will be supervised by the project leader. At each supervision, participants will be required to stay in the hospital for 30 minutes after treatment and asked about any signs of acute adverse reactions during the study. Side effects will be recorded at every visit during treatment.

### 2.2 Sample size calculation

#### 2.2.1 Statistical analysis

Trained statisticians will use intentional processing methods for effectiveness and safety analysis. In addition, the final outcome observation index method will be applied to deal with missing values. All statistical analysis will be performed by the SPSS 21.0. The statistical test is two-way, *P* < .05 is statistically significant. The mean ± standard deviation will be used to describe continuous variables. This percentage is used to describe the variable. Continuous variables that follow a normal distribution will be analyzed by Student *t* test; otherwise, nonparametric tests will be used to compare group differences.

#### 2.2.2 Data collection and monitoring

This is a 4-week clinical trial. Participants need to conduct a 4-week research intervention during a 4-week follow-up. Five rounds of disease activity assessment (baseline and week 1, 2, 3, and 4) will be independently recorded by 2 investigators in Epidata (version 3.1), and differences will be resolved through discussion and a third research institute. Longhua Hospital affiliated to Shanghai University of Traditional Chinese Medicine is responsible for monitoring and quality control.

#### 2.2.3 Quality control

Before conducting the study, we will conduct unified training to ensure that all relevant doctors, nurses and evaluators fully understand the entire trial process. In order to ensure the quality of the entire study, 2 supervisors will be sent every month to ensure that the enrolled patients meet the inclusion criteria and do not meet the exclusion criteria; the research group recruits enough patients to participate in the study; all enrolled patients fully follow the clinical trial Process, perfect the case report form (CRF), follow the standard operating procedure (SOP). During the entire treatment and follow-up period, the inspector will record the dropout, withdrawal, and related reasons in detail, and record any compliance of all patients.

## 3 Discussion

The clinical features of CSR are that the pain range is consistent with the compressed spinal nerve area, mainly pain, numbness, and hypoesthesia. In severe cases, radicular weakness, and muscle atrophy may occur. After the occurrence of CSR, a large number of inflammatory factors are released from the damaged tissue, which stimulates the nerve root and causes radicular pain symptoms.^[13]^ At the same time, it activates a variety of cell signal transduction pathways, cytokines and receptor proteins in the body, which in turn can induce interleukin-1β (IL-1β), interleukin-6 (IL-6), and tumor necrosis factor- α (TNF-α) and other pro-inflammatory cytokines are secreted to mediate inflammation and form a vicious circle of inflammation.^[13]^ The heat generated by herb compresses causes blood vessels to dilate and muscles relax. Heat therapy is a common alternative therapy to improve blood circulation and local muscle spasms. The various ingredients of herb have a variety of analgesic and anti-inflammatory properties. The herb in this research contain Corydalis, Yang Bin, etc.^[14]^ When studying the extracts of Chinese medicine Corydalis Corydalis, they found that Corydalis extracts can exert the same effect as NLRP3 inhibitors. Inhibit NLRP3 inflammasome signaling pathway protein expression. The NLRP3 inflammasome signaling pathway can be widely involved in the body's inflammatory response and promote the synthesis and secretion of inflammatory factors such as prostaglandin E2 (PGE2), phospholipase A2 (PLA2), IL-6, and TNF-α.^[15]^ Corydalis extract inhibits NALP3 and then inhibits the inflammatory response, relieves the inflammatory response caused by nerve root compression, and reduces secondary spinal cord injury.

Traditional Chinese medicine can not only inhibit nerve root inflammation, relieve nerve root edema, and fundamentally alleviate the root symptoms of CSR, but also can increase the body's pain threshold, improve the body's spontaneous pain and hyperalgesia caused by inflammatory stimulation,^[16]^ and relieve it quickly. The first symptoms of CSR are contradictory. The active ingredient xanthamide extracted from Zanthoxylum bungeanum can significantly increase the pain threshold of the CSR body, so that the body can withstand greater intensity of pain stimulation. Moreover, prickly amide can reduce the levels of inflammatory factors TNF-α, IL-1β, and cyclooxygenase-2 (COX-2), play an anti-inflammatory and analgesic effect, and have a certain control effect on the symptoms of CSR [24]. The active ingredient ferulic acid (FA) extracted from Ligusticum chuanxiong can also increase the pain threshold of the CSR body, and Zhao Weihong and others have observed that FA can significantly improve the obvious edema and degeneration of nerve root tissue and demyelination caused by lesions. To improve the potential motor conduction velocity (MCV) of the median nerve of the CSR body.^[17]^

Therefore, we plan to conduct this prospective randomized controlled clinical trial to study the clinical efficacy of traditional Chinese medicine hot compress combined with traction in the treatment of cervical spondylotic radiculopathy, and to explore how to use the most combination of conservative therapies to improve cervical pain and cervical radiculopathy. Radical symptoms and explore whether it can be the first choice for potential conservative treatment of CSR.

## Author contributions

Xing Ding, Jinze Wu are co-first authors of this manuscript, contributing equally to the design, conduct of the trials, and drafting of the manuscript. All authors participated in the design of the study and performed the trial. Jinhai Xu and Wen Mo are the co-corresponding authors of this manuscript, contributing equally to the supervision and coordination of the clinical trial. All authors read and approved the final manuscript.

**Conceptualization:** Xing Ding, Jinze Wu, Qixing Shen, Wen Mo.

**Data curation:** Xing Ding, Jinze Wu, Jinhai Xu, Wen Mo.

**Formal analysis:** Xing Ding, Jinze Wu, Qixing Shen, Jinhai Xu, Wen Mo.

**Funding acquisition:** Xing Ding, Jinze Wu, Qixing Shen, Jinhai Xu, Wen Mo.

**Investigation:** Xing Ding, Jinze Wu, Qixing Shen, Jinhai Xu.

**Methodology:** Xing Ding, Jinze Wu, Qixing Shen.

**Project administration:** Xing Ding, Qixing Shen, Jinhai Xu, Wen Mo.

**Resources:** Xing Ding, Qixing Shen, Jinhai Xu, Wen Mo.

**Software:** Xing Ding, Jinze Wu, Jinhai Xu, Wen Mo.

**Supervision:** Xing Ding.

**Validation:** Xing Ding, Jinhai Xu, Wen Mo.

**Visualization:** Xing Ding, Jinze Wu, Jinhai Xu.

**Writing – original draft:** Xing Ding, Jinze Wu, Jinhai Xu, Wen Mo.

**Writing – review & editing:** Xing Ding, Jinze Wu.

## Abbreviations

Abbreviations: COX-2 = cyclooxygenase-2, CRF = case report form, CSR = cervical spondylotic radiculopathy, FA = ferulic acid, IL-1β = interleukin-1β, MCV = motor conduction velocity, NDI = Neck Disability Index, TNF-α = tumor necrosis factor- α, VAS = Visual Analog Scale.
